# Dataset of operational energy of forty Andean buildings from 1980 to 2020

**DOI:** 10.1016/j.dib.2025.111818

**Published:** 2025-06-22

**Authors:** Jefferson Torres-Quezada, Atila Avila-Argudo

**Affiliations:** a CONSISOlab, School of Architecture, Universidad Católica de Cuenca, Ave. Americas & General Torres Engineering building, 1st floor, Cuenca 010102, Ecuador; b CONSISOlab, Center of Innovation, Research and Transferring of Technology (CIITT), Universidad Católica de Cuenca, Ave. to Bibin, Ricaurte, Cuenca 010108, Ecuador

**Keywords:** Space heating, Space cooling, Construction materials, Building simulation

## Abstract

This dataset presents operational energy data from forty residential buildings constructed between 1980 and 2020 in Cuenca, a city in the Andean region of Ecuador. It includes energy consumption data related to heating, cooling, lighting, electrical appliances, domestic hot water and cooking. Ten sample houses from each decade were selected, representing typical construction practices of their respective periods. The study follows three main stages: (1) Analysis of operational energy consumption, showcasing the evolution of energy use across four decades; (2) Simulation and validation, where energy simulations and calculations are performed for each sample, followed by a validation process using *in-situ* measurements compared with simulated results; and (3) Data curation, where climate data is compiled and updated for further analysis. This dataset includes files and figures that enhance comprehension and support further research on energy efficiency, sustainable building design, and energy policy development in regions with moderate climates. It also enables comparisons with datasets from other geographic regions, contributing to a broader understanding of energy demand patterns in residential buildings.

Specifications Table

**Table:** 

Subject	Engineering & Materials science
Specific subject area	The research addresses the area of architecture design from the perspective of the life cycle energy of buildings, focusing on the Operational Energy.
Type of data	Tables (xlsx), Figures (.png), Raw data from temperature and climate measurements, Analyzed data (Design Builder Files).
Data collection	The data for operational energy was gathered in two different instances. First, for Space Heating plus Space Cooling and Lighting plus Electrical Appliances, digital simulations were undergone using Design Builder as software and Energy Plus as the calculation engine. Building features were based upon planimetric models and occupancy surveys performed *in-situ*. To validate the simulation model, eight houses measured on site were compared to their corresponding simulations. In second place, for Domestic Hot Water plus Cooking, a common standardized value was stablished, derived from calculations referred to relevant bibliography related to the consumption of these uses in Ecuador.
Data source location	Cuenca, Ecuador
Data accessibility	Repository name: Mendeley Data identification number: DOI:10.17632/jt9kc43ttg.2 Direct URL to data: https://data.mendeley.com/drafts/jt9kc43ttg
Related research article	Jefferson Torres-Quezada, Antonio Isalgue-Buxeda, Anna Pages-Ramon, Judit Lopez-Besora, Operational and embodied energy of residential buildings in the Andean region between 1980 and 2020, (2024) https://doi.org/10.1016/j.enbuild.2024.114982

## 1 Value of the Data

• This dataset provides detailed information on the operational energy consumption of 40 residential buildings in Cuenca, Ecuador, spanning the last four decades (1980–2020). It offers a broad sample of information in a region where such data is often scarce or unavailable. The data presented in excel format (.xlsx) includes energy consumption data for space heating and Space cooling (sc + sh), lighting and electrical appliances (l + e), and domestic hot water plus cooking (d + c). The first two operational energy groups were estimated through simulations and validation processes based on detailed information collected on-site. While the third group was calculated through mathematical calculations, deriving into a standardized value that was used for all the samples, based on relevant bibliographic sources [1].
• Given the large and diverse amount of housing information, researchers can use this dataset to analyze the relationship between morphological and material characteristics of buildings and their energy consumption, contributing to a deeper understanding of energy demand patterns in residential architecture of this climate.
• Additionally, the dataset includes configured Design Builder (.dsb) files for all forty houses, providing significant value for further analysis. These files are fully structured with morphological and material characteristics, as well as intrinsic data embedded within the Design Builder software. This allows researchers to conduct additional simulations, modify parameters, and explore energy variations with high accuracy. Moreover, the files allow for the assessment of key building characteristics—such as window-to-wall ratio (WWR), orientation, and energy consumption impact—as well as their influence on occupant thermal comfort over time. For instance, a research group addressing the thermal behavior and energy consumption of a house in this Andean region, could use the .dsb files as templates to skip time consuming processes regarding this analysis.
• The dataset includes weather station data as well as data measured *in-situ* (.epw). This data is related to climate aspects and indoor thermal comfort respectively; these can serve as a foundation for the calibration and validation of energy simulations. Moreover, this data is particularly useful for energy efficiency studies and for evaluating correlations between operational energy and other architectural variables.
• This dataset can serve as a theoretical basis for municipal regulatory departments to implement regulations aimed at the energy efficiency of Andean buildings such as Quito, Ambato and Cuenca referenced in [[1], [2], [3]]. Its applicability may also extend to other cities across the Andean region of South America. Furthermore, research centers at universities located in these areas could benefit from this data article by supporting studies related to operational energy performance such as Renewable Energies Lab [4], Llacta lab [5] and others.

## 2 Background

The motivation behind compiling this dataset was to see the trend in the changes of OE in the evolution of the construction systems used in Andean residential buildings over the last four decades (1980 to 2020). To analyze this evolution, ten residential buildings from each decade were selected. To obtain the operational energy for each sample, data was collected on all homes *in situ*, which was used to configure and validate the simulation models. This data, yet related to the original research article named “Operational and Embodied Energy of Residential Buildings in the Andean Region between 1980 and 2020” [6], emphasizes in compiling wider and more specific firsthand sources, which foster future research in this field.

The information needed to obtain the operational energy of these forty buildings was extensive and detailed; however, it was summarized in the original article. Thus, this data in brief article aimed to provide researchers with a comprehensive resource for analyzing operational energy (OE) consumption in residential buildings. This dataset complements the original research article, providing detailed and validated data from all analyzed samples, which can be used for further analysis and comparison.

Additionally, the dataset supports research on energy demand trends and their implications for sustainable development in Andean climates. It provides foundational data for calibrating energy models, benchmarking efficiency standards, and exploring the relationship between building design and energy performance. By offering high-quality, structured data, this resource aims to facilitate further research in operational energy within residential architecture and contribute to broader investigations in the global scientific community.

## 3 Data Description

The files presented in this Data-in-Brief Article are divided into three sections: Operational Energy Data, Simulation Data and Climate Data. The repository is structured accordingly. Therefore, the first folder 01_OperationalEnergyDATA, contains a file named STOE.xlsx, which summarizes the operational energy data of the 40 sample houses. The second folder 02_SimulationDATA, contains 4 subfolders P1, P2, P3 and P4, each one of them allocates the ten simulation files (.dsb) corresponding to each sample and period (P). Moreover, the second folder contains three excel files, Building_Features.xlsx, Measuraments&Simulations_Results.xlsx and Measurements.xlsx, all of them related to morphological/materials characteristics and validation and simulation reports of each sample. Finally, the third folder 03_ClimateDATA, contains two files, Climate_Data_Forupdate.xlsx and ECU_CUENCA-_WEC_2022.epw, both related to the climate data needed for simulations. The detailed review of each section is described below:

### 3.1 Operational energy data

The first part of the data is the entire house Operational Energy, named as Specific Total Operational Energy STOE, which accounts for the summatory of space heating and space cooling (sh + sc), lighting and electrical appliances (l + e) and Domestic Hot Water and Cooking (d + c) The values ​​for the first two groups were obtained from the specific characteristics of each sample home, while, the last group was addressed through standardization processes, related to relevant bibliography to get a standardized value for all samples-, then, the summatory of these energies are divided by the total floor area of each residential building. The STOE is calculated for each of the forty houses in the study accounting both annually and a 50-years lifespan consumption.

**Fig. 1:**
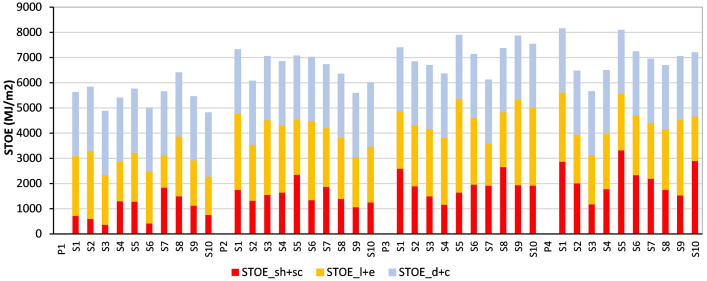
STOE 50-years lifespan of all samples in each period divided in STOE_sh+sc, STOE_l+e and STOE_d+c.

Fig. 1 illustrates the Specific Total Operational Energy for all analyzed samples across the four periods, divided in the three groups: sh+sc, l+e and d+c. This figure shows the 50-years lifespan values presented in MJ/m².

These results are shown in the repository in the file named STOE.xlsx. This data is structured as shown in Table 1.

**Table 1:** STOE.xlsx file structure.

a)	b)	c)	d)	e)	f)	g)	h)	i)	j)	k)	l)	m)	n)	o)	p)
Period and Sample	Total Floor Area (TFA) (m2)	Space Heating (sh) (kWh)	Space Cooling (sc) (kWh)	(sh + sc) (kWh)	Lighting (l) (kWh)	Electrical Appliances (e) (kWh)	Total (l + e) (kWh)	(sh + sc) (kWh/m2)	(l + e) (kWh/m2)	(d + c) (kWh/m2)	(sh + sc) 50 Years (MJ/m2)	(l + e) 50 Years (MJ/m2)	(d + c) 50 Years (MJ/m2)	Annually STOE (kWh/m2)	50 years STOE (MJ/m2)
P1_S1	347	1403.1	1.39	1401.71	935.61	3637.25	4572.86	4.04	13.18	13.98	727	2372	2516	31.2	5616
P1_S2	281	948.34	3.09	951.43	1259.21	2950.05	4209.26	3.39	14.98	13.98	609	2696	2516	32.35	5823
P1_S3	283	588.35	5.61	593,96	725.77	2383.34	3109.11	2.1	10.98	13.98	378	1978	2516	27.06	4870.8
P1_S4	270	1940.87	22.1	1962.97	947.66	1412.88	2360.54	7.27	8.74	13.98	1309	1574	2516	29.99	5398.2
P1_S5	192	1370.91	5.95	1376.86	476.6	1594.63	2071.23	7.17	10.79	13.98	1291	1942	2516	31.94	5749.2

In reference to this table, columns a) and b) contain the information regarding the building's identification code and the surface area respectively. The a) column is named Period and Sample and presents the buildings code where each one of the houses are labelled with the period and sample number as P1_S1, P2_S2...P4_S10 respectively. The second column presents the total floor area (TFA) in m^2^ of the respective house. The TFA refers to the sum of the area of each floor of the building measured to the outer surface of the exterior walls excluding the garage, front/back yard, and gardens.

The following six columns from c) to h) contain data regarding the measured energy per sample in kWh of space heating and cooling (sh + sc) and lighting plus electrical appliances (l + e). The column c) presents the space heating energy (sh), the column d) shows the space cooling energy (sc). The column e) presents the summatory of these last two as (sh + sc). The column f) presents the energy consumed by lighting (l), the column g) presents the energy consumed by electrical appliances (e). The column h) presents the summatory of these last two energies (l + e).

Column i) and k) contain the data from the previous energy consumed totals in kWh (column e and h) divided by the total floor area in m^2^ (column b). The values regarding (sh + sc) are presented in column i) and the values correspondent to (l + e) in column j). Besides, the column k) shows the value of (d + c), this last was acquired throughout mathematical calculations from related bibliography so this value is common for all the samples analysed. The data in this section is presented in kWh/m2

Columns m), n) and l) show the values of the prior section respectively but extrapolated to a 50-year lifespan and presented in MJ/m2. The last two columns o) and p) present the total STOE (sh+sc+i+e+d+c) expressed for an entire year in kWh/m2 and for a 50-year lifespan in MJ/m2 respectively.

### 3.2 Simulation data

This section contains two types of files. On one hand, numerical data is presented in three Excel files which are in the repository with the names: Building_Features.xlsx, Measurements.xlsx, and Measurements&Simulations_Results.xlsx. On the other hand, there are forty Design Builder (.dsb) files which are in the repository in a folder named 02_SimulationDATA. All these files are explained in detail as follows:

In First instance this section presents the Building_Features.xlsx table that contains the raw data from the envelope materials of each one of the 40 sample buildings. This information was collected *in-situ* according to the structure shown in Table 2. The first column specifies the period and the sample. The following five columns show: the total floor area, number of floors, Window-to-wall ratio, U-value of wall and U-value of roof. The next columns show the materials used in walls, roofs and windows.

**Table 2:** Building_Features.xlsx file structure.

Period & Sample	TFA (m2)	No. Floors	WWR (%)	U_wall (W/m^2^.K)	U_roof (W/m^2^.K)	WALLS					ROOF					WINDOW			
						Solid brick 30 cm	Solid brick 15cm	Hollow brick 15 cm	Hollow pumice block 15 cm	Cement mortar plaster	Fiber cement	Concrete slab	Air chamber +ceiling	One layer of tile	Two layers of tile	Single glass	Steel frame	Wood frame	Aluminum frame
P1_S1	347	2	28%	1.649	1.037	x				x	x		x		x	x	x		
P1_S2	281	2	25%	1.649	1.037	x				x	x		x			x	x		
P1_S3	283	2	23%	1.649	1.037	x				x	x		x		x	x	x		
P1_S4	270	2	35%	2.165	1.552		x			x	x		x		x	x	x		
P1_S5	192	2	31%	2.461	1.552		x			x	x		x		x	x		x	

Furthermore, the Measurements.xlsx file contains the raw data measured *in situ* from eight houses used in the study to validate the simulations, these measurements are expressed in°C. The structure of the table is shown in Table 3. The first two columns show the date and the time of each measurement taken in 5-minutes interval. The following columns show the indoor temperature of two samples of each period. The samples measured are sample 3 and 10 from period 1, sample 1 and 5 from period 2, sample 4 and 8 from period 3 and sample 8 and 10 from period 4.

**Table 3:** Measurements.xlsx file structure.

Date	Time	Ti_P1_S3 °C	Ti_P1_S10 °C	Ti_P2_S1 °C	Ti_P2_S5 °C	Ti_P3_S4 °C	Ti_P3_S8 °C	Ti_P4_S8 °C	Ti_P4_S10 °C
7/7/2021	11:15:00 PM	17.9	18.6	18.2	18.3	18.2	19.9	17.3	18.6
7/7/2021	11:20:00 PM	17.9	18.6	18.3	18.3	18.2	19.8	17.2	18.2
7/7/2021	11:25:00 PM	17.9	18.6	18.3	18.3	18.2	19.8	17.2	18.1
7/7/2021	11:30:00 PM	17.9	18.6	18.3	18.3	18.2	19.8	17.2	18.1
7/7/2021	11:35:00 PM	17.9	18.6	18.3	18.3	18.2	19.8	17.2	18.1

In regard to the Measurements&Simulations_Results.xlsx file, this table contains the raw data from each one of the eight samples measured *in-situ* and simulated in DesignBuilder accordingly. The structure of this data is shown in Table 4. The first two columns show the date and the time of each measurement taken in 1-hour interval. The following columns show the measured temperature data after the simulated data of their respective sample; these measurements are expressed in°C. Additionally, the last columns show the Root Square Mean Error of each sample. Table 4 presents the first twelve columns of the file solely, to see the entire data refer to the repository where the main file containing all the remaining information can be found.

**Table 4:** Measured_Simulated.xlsx file structure.

Date	Time	M_P1_S3 °C	S_P1_S3 °C	M_P1_S10 °C	S_ P1_S10 °C	M_P2_S1 °C	S_P2_S1 °C	M_P2_S5 °C	S_P2_S5 °C	M_P3_S4 °C	S_P3_S4 °C
8/7/2021	0:00	18	17	17	18	18	17	17	18	18	18
8/7/2021	1:00	18	17	17	18	18	17	17	18	18	18
8/7/2021	2:00	18	18	17	18	18	17	17	18	17	18
8/7/2021	3:00	18	18	17	18	17	17	17	18	17	17
8/7/2021	4:00	18	17	17	18	17	17	17	18	17	17

**Fig. 2:**
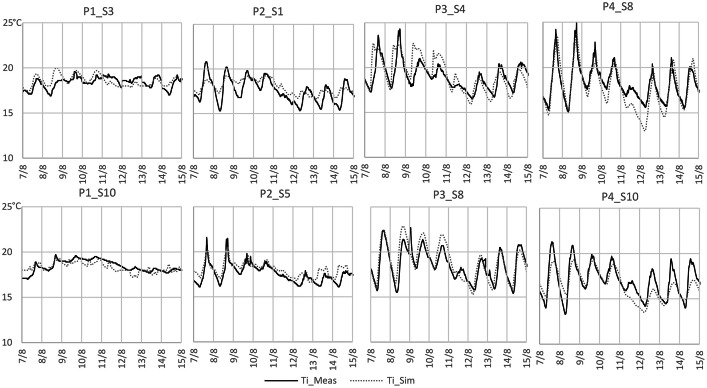
Graphical comparison between Indoor temperature of 2 samples of each period obtained by simulations (TI_sim) and *in situ* measurements (Ti_meas).

Fig. 2 shows the graphical comparison between simulations and measurements results of all samples.

Lastly, this section presents 40 Design Builder Files (.dsb) one for each of the 40 analysed houses and stored in separate .dsb files, in four folders P1, P2, P3 and P4 for each one of the four decades accordingly. The files inside the forlders are named using the format P#_S#, where P represents the period (decade), and S represents the sample number. Each file is configured according to the unique morphology and the materials are taken from the file Building_Features.xlsx explained at the beginning of this section. The intensity and schedule of lighting, equipment and occupancy were established as a common setting for all forty samples. This setting is detailed in the method section.

### 3.3 Climate data

This section contains two files. The first one is the climate data gathered to update the weather file to use for simulation, named as *Climate_Data_Forupdate.xlsx.* The second one is the updated climate file itself, named as *ECU_CUENCA_IWEC_2022.epw.*

**Fig. 3:**
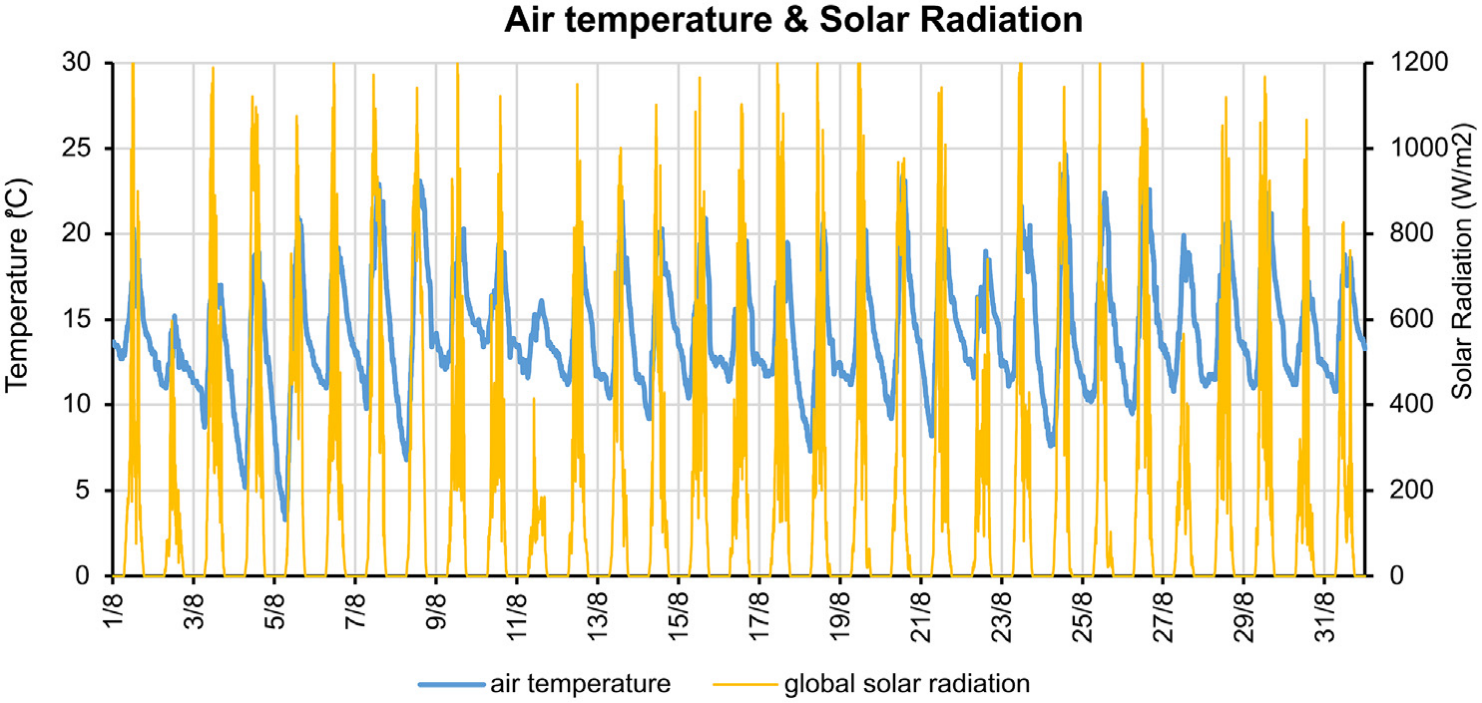
Air temperature & solar radiation.

This first file *Climate_Data_Forupdate.xlsx* is an excel table, which was created through the climate data gathered during July and August from the weather station ICUENC29 (coordinates: 2.89°S, 79.01°W, altitude: 2564 masl) to update the EPW (Energy Plus Weather) file for the simulation software. This information was taken from [7]. The climate factors collected and registered in this file are: date & time, air temperature, dew point, relative humidity, wind direction, wind speed, gust speed, atmospheric pressure, rain, global solar radiation. The air temperature and solar radiation values gathered in these measurements are shown in Fig. 3.

Finally, the second file *ECU_CUENCA_IWEC_2022.epw is* an Energy Plus Weather file which was updated with the information collected from the meteorological station from July 1, 2021, at 12:04 AM to August 31, 2021, at 11:59 PM, with a sampling interval of 1-hour. This file was used in the simulations of all samples to obtain the operational energy for sh+sc and l+e, as explained before.

## 4 Experimental Design, Materials and Methods

To calculate the Specific Total Operational Energy (STOE) for each of the 40 residential building samples, the study was designed around four distinct periods, with each period comprising 10 sample houses. These periods are P1 from 1980 to 1990), P2 from 1991 to 2000, P3 from 2001 to 2010 and P4 from 2011 to 2020, corresponding to the last four decades accordingly. First, a selection process was carried out to obtain the 40 samples. This process began by identifying urban sectors with recorded samples from four periods. From these sectors, 61 residential buildings were randomly pre-selected based on their construction year, using the city's municipal cadaster. However, 21 buildings were excluded due to accessibility issues and missing technical data. Ultimately, 10 residential buildings per period were chosen. Once the final sample was established, the official architectural plans of the homes were compiled. However, in cases (13 samples) where the owners did not have these plans, an on-site planimetric survey was conducted to obtain the morphological and material characteristics of the homes, in conjunction with verification by the owners. Furtehrmore, the operational energy uses considered were space heating and space cooling (sh + sc), lighting and electrical appliances (l + e), and domestic hot water and cooking (d + c). The (sh + sc) and (l + e) components were derived from simulation results, whereas the (d + c) component was standardized by calculations using established mathematical methods from the literature.

For the simulation of the (sh + sc) and (l + e) components, all forty sample houses were modeled in Design Builder software, with simulations executed in Energy Plus. The morphological characteristics and material specifications of each sample were configured based on on-site data collection and architectural plans. In addition, information on occupancy and space usage was compiled to develop a common model reflecting the most frequent schedules observed among the samples [6], derived from a survey carried out *in situ*.

This occupancy survey was conducted only with the owners of each home in the sample. The process consisted of completing a form detailing the approximate occupancy times of each space on weekdays and weekends, as well as the lighting and electrical appliances. The spaces of a sample house were standardized in: Sitting Room, Study Room, Children Bedroom, Master Bedroom, Kitchen, Dining Room and Living room, during weekdays and weekends.

Table 5 provides an overview of the occupancy, climatization, lighting, and electrical appliance values and schedules configured for the residential buildings.

**Table 5:** Occupancy, climatization, lighting, and electrical appliances values and schedules configured for the 40 residential buildings. Source: Ref. [6].

Space	Occupancy (people/m2)	Occupancy/ Climatization Schedule (Weekdays)	Occupancy/ Climatization Schedule (Weekend)	Lighting Schedule (Weekdays)	Lighting Schedule (Weekend)	Electrical Appliances Schedule (Weekdays)	Electrical Appliances Schedule (Weekend)
Sitting room	0.03	_	14h - 19h	_	18h - 19h	_	14h - 19h
Study room	0.06	_	14h - 19h	_	18h - 19h	_	14h - 19h
Children bedroom	0.05	00h - 07h	00h - 07h	20h - 22h	20h - 22h	20h - 00h	08h - 13h
		20h - 00h	08h - 13h				14h - 19h
			14h - 19h				20h - 00h
			20h - 00h				
Master bedroom	0.1	00h - 07h	00h - 07h	20h - 22h	20h - 22h	20h - 00h	20h - 00h
		20h - 00h	20h - 00h				
Kitchen	0.04	07h - 08h	07h - 08h	19h - 20h	19h - 20h	07h - 08h	07h - 08h
		19h - 20h	13h - 14h			19h - 20h	13h - 14h
			19h - 20h				19h - 20h
Dinning room	0.03	07h - 08h	07h - 08h	19h- 20h	19h - 20h	07h - 08h	07h - 08h
		19h - 20h	13h - 14h			19h - 20h	13h - 14h
			19h - 20h				19h - 20h
Living room	0.03	_	13h - 18h	_	_	_	13h - 18h

Within the simulation environment, key parameters were established as follows:

• Indoor Comfort Range: Set between 17.62°C and 22.62°C, based on Ref. [8].
• The spaces accounted for climatization are the kitchen, living room, dining room, sitting room, study room and bedrooms
• Simulation Duration: A full-year simulation period was applied to each sample.

Once these parameters were configured, simulations were run for one full year, with the results documented in the file *Measurements&SimulationsResults.xlsx*.

Before the simulations of all samples, the model was validated against *in situ* measurements. Two representative building samples from each period (totaling eight samples) were selected for monitoring. These buildings were monitored from August 3, 2021, at 8:18 PM to August 28, 2021, at 4:55 AM, with data recorded at five-minute intervals. Concurrent climate data was also collected during this period.

Measurements were performed using a TESTO 174H Sensor, with its specifications detailed in Table 6. The raw measurement data is available in the repository as *Measurements.xlsx*. Updated climate data— Using a weather station [7] recorded from a from August 1, 2021, at 12:04 AM to August 31, 2021, at 11:59 PM with a five-minute sampling interval—is stored in the repository as *Climate_Data_Forupdate.xlsx*. Relevant data from these measurements were extracted to update the final climate file, stored as *ECU_CUENCA_IWEC_2022.epw*, which was later used in the simulation models.

**Table 6:** Technical specifications of testo 174H.

Characteristic	Values
Measuring range	0 to 100% RH (non-dewing atmosphere),-20 to +70°C
Humidity accuracy	± 3% RH (2% RH to 98% RH) ± 1 digit +0.03% RH/K)
Temperature accuracy	± 0.5°C(-20 to +70°C)
Resolution	0.1% RH, 0.1°C
Operating temperature	from -20 to +70°C
Operating manual	https://media.testo.com/media/47/aa/d7688350fbfd/testo-
Software	174_Instruction-Manual.pdf

Subsequently, simulations for the eight sample houses were configured in Design Builder [9] based on their morphology, material properties, and space usage, and executed using Energy Plus [10]. The resulting simulation data were compared with the corresponding *in-situ* measurements for each house (see Fig. 2).

Once the simulation model was validated, simulations were run for the 40 sample homes according to the specifications explained above. The simulation files for each of the analyzed samples are available in the repository.

Finally, for component (d + c) energy consumption was obtained using calculations based on the estimated consumption given by [11] which specifies that a residential building consumes 18.45 kg of LPG per month. This estimation was transformed into energy using the calorific value of LPG (13.39 kWh/kg) [12]. This value was converted to MJ and multiplied by twelve to determine the annual STOE for the (d + c) component. Finally, this number was divided by the average total floor area of the forty buildings analyzed. Because of this, operational energy for (d + c) has the same value for all samples.

The total annual STOE was then computed by summing the operational energy values for (sh + sc), (l + e), and (d + c). Additionally, a STOE value for a 50-year building lifespan was calculated to provide an estimation of the life cycle operational energy of the sample houses. These results are presented in the file *STOE.xlsx*.

## Limitations

The dataset presented in this study has several limitations that should be considered. First, the sample size is constrained due to the lack of publicly available morphological data for residential buildings. As a result, each house had to be individually measured and modeled, requiring significant time and resources. This labor-intensive process necessitated a practical limit on the number of samples included, though future studies could benefit from a broader dataset.

Second, some data was based on mathematical estimations, like d + c. These assumptions were necessary for consistency in modeling, since this standardized value was used to set the simulation for the 40 samples, although this may limit the applicability of the findings to scenarios involving non-standard energy consumption behaviors.

Finally, the dataset focuses on residential buildings in the Andean region of Ecuador, excluding data from other climatic zones, such as the Costa region. This geographic limitation affects the generalizability of the results across different Ecuadorian climates and building typologies. Expanding the dataset to include diverse climatic conditions would enhance its relevance for nationwide energy analyses.

Despite these constraints, the dataset offers valuable insights into operational energy consumption in Ecuadorian residential buildings and provides a strong foundation for further research on energy efficiency and sustainability in similar contexts.

## Ethics Statement

The authors have read and followed the ethical requirements for publication in Data in Brief and confirming that the current work does not involve human subjects, animal experiments, or any data collected from social media platforms.

## CRediT authorship contribution statement

**Jefferson Torres-Quezada:** Conceptualization, Methodology, Investigation, Writing – review & editing, Supervision. **Atila Avila-Argudo:** Writing – original draft.

## Data Availability

(Mendeley Data).Dataset of Operational Energy of Forty Andean Buildings from 1980 to 2020 (Original data)
